# Subclinical Mastitis in Small-Holder Dairy Herds of Gansu Province, Northwest China: Prevalence, Bacterial Pathogens, Antimicrobial Susceptibility, and Risk Factor Analysis

**DOI:** 10.3390/microorganisms12122643

**Published:** 2024-12-20

**Authors:** Ling Wang, Shahbaz Ul Haq, Muhammad Shoaib, Jiongjie He, Wenzhu Guo, Xiaojuan Wei, Xiaohong Zheng

**Affiliations:** 1 Key Laboratory of New Animal Drug Project, Gansu Province, Lanzhou 730050, China; wangling02@caas.cn (L.W.); guowenzhu1980@163.com (W.G.); wxjsqss@163.com (X.W.); 2018593015@st.gxu.ed.cn (X.Z.); 2 Key Laboratory of Veterinary Pharmaceutical Development, Ministry of Agriculture and Rural Affairs, Lanzhou 730050, China; 3 Lanzhou Institute of Husbandry and Pharmaceutical Sciences of Chinese Academy of Agriculture Sciences, Lanzhou 730050, China; 4 Department of Pharmacology, Shantou University Medical College, Shantou 515041, China; shahbazulhaq.vet@gmail.com; 5 Jiangsu Co-Innovation Center for Prevention and Control of Important Animal Infectious Diseases and Zoonoses, College of Veterinary Medicine, Yangzhou University, Yangzhou 225012, China

**Keywords:** subclinical mastitis, risk factor, etiology, occurrence, antimicrobial resistance

## Abstract

This cross-sectional study assessed the prevalence, bacterial distribution, antimicrobial susceptibility, and potential risk factors associated with subclinical mastitis (SCM) in small-holder dairy herds in Gansu Province, Northwest China. Forty small-holder cow farms were randomly selected from eight cities/counties in six districts of Gansu Province, and a total of *n* = 530 lactating cows were included in this study. SCM prevalence was noted at 38.87% and 9.72% at the cow and quarter levels, respectively, based on the California Mastitis Test (CMT). The prevalence of the recovered bacterial species was noted as follows: *S. agalactiae* (36.02%), *S. aureus* (19.43%), coagulase-negative staphylococci (CNS) (16.11%), *S. dysgalactiae* (12.80%), *E. coli* (9.00%), and *S. uberis* (6.64%). All isolated bacteria were 100% multi-drug-resistant (MDR) except *S. aureus* (87.8% MDR). Antimicrobial susceptibility profiles revealed the increased resistance (>85%) of these pathogens to penicillin, streptomycin, trimethoprim–sulfamethoxazole, vancomycin, and erythromycin. However, these pathogens showed increased susceptibility to ampicillin, amoxicillin–sulbactam, ceftazidime, neomycin, kanamycin, spectinomycin, norfloxacin, ciprofloxacin, and doxycycline. The multivariate regression analysis demonstrated that old age, high parity, late lactation, lesions on teats, previous history of clinical mastitis, higher milk yield, and milking training were found to be potential risk factors (*p* < 0.001) associated with developing SCM in small-holder dairy cows in Gansu Province, China. These findings highlight the need for routine surveillance, antimicrobial stewardship, and effective preventive strategies to mitigate SCM in small-holder dairy production and their possible impacts, i.e., increased antimicrobial resistance and infection, on public health.

## 1. Introduction

Mastitis is defined as the inflammation of the mammary gland considered the most costly and prevalent disease in dairy production. Mastitis is categorized into two primary forms: clinical and subclinical. Clinical mastitis (CM) is defined by clinical signs and symptoms such as abnormal milk, lower milk production, udder swelling, the discharge of blood from teats, etc. [1]. Subclinical mastitis (SCM) is considered the silent form of mastitis. It is responsible for increased losses because of the lack of clinical signs, its increased prevalence relative to clinical mastitis (CM), and its association with potential risk factors [2,3]. SCM results in discarded milk and reduced milk production, accounting for approximately 20–60% of the costs associated with SCM [4]. The most frequent microorganisms responsible for causing mastitis include contagious pathogens (e.g., *Streptococcus agalactiae* and *Staphylococcus aureus*) and environmental pathogens (coagulase-negative staphylococcus (CNS), *Streptococcus dysgalactiae*, and coliforms) [5,6,7,8]. Contagious pathogens are considered predominant organisms that have adapted to survive within the mammary gland of the host and are capable of causing the development of subclinical infections. They can spread within a flock, resulting in an epidemic situation [6]. Moreover, various predisposing factors have been identified, such as lactation period, parity number, health status, milking practices, hygienic measures, and previous antibiotic usage [7]. The extensive use of antibiotics in farm animals may lead to the emergence of multi-drug-resistant bacteria in the environment and, ultimately, transfer to human settings, resulting in treatment failure in clinical settings [9,10]. Previous studies conducted on large-scale dairy farms in various regions of China recorded an average pooled SCM prevalence of 37.7% [11]. Moreover, antimicrobials were used extensively in the dairy industry to prevent and control mastitis and other bacterial diseases affecting dairy cows. SCM is a significant issue that compromises the profitability of the dairy industry in China, and dependency on antimicrobials has become a common phenomenon on dairy farms [12]. The California Mastitis Test (CMT) is considered the most convenient, sensitive, and reliable method for detecting SCM [13] compared to somatic cell count (SCC). However, SCC is regarded as one of the laboratory tests used to control SCM, and an SCC > 200,000 cells/mL indicates positivity for SCM [14]. However, a study conducted by Abed et al. [15] in Egypt compared the prevalence of SCM based on the CMT (46%) and SCC (44%) and did not find a significant difference between them. Moreover, the CMT’s sensitivity, specificity, and accuracy are 86.10%, 59.70%, and 75.50%, respectively [16]. Thus, detecting SCM through the CMT is helpful at a small scale and gives early signals before CM develops.

The total dairy cow population in Gansu Province is estimated to be 200,000, and the Chinese Holstein cow breed comprises the majority of dairy cows. Small-holder dairy farms (≤100 lactating cows per farm) account for 40.02% of the total dairy cows in Gansu Province and approximately 24.21% of the total raw milk production in this province [17], which is also a significant source of income for rural communities. Herd management in large-scale dairy farms cannot be generalized to small-holder dairy farms. Few investigations have recorded the potential risk factors and predominant bacterial pathogens associated with SCM in local small-holder dairy farms. Monitoring antimicrobial susceptibility in pathogens is essential for animal and public health and provides necessary information for outreach, advice, and policy recommendations. This study aimed to identify the distribution and antimicrobial resistance (AMR) profile of predominant pathogens in SCM of small-holder dairy cows to provide proper guidelines for the correct antibiotic selection. The present study also identified the potential risk factors of SCM in small-holder dairy cows through multivariate regression analysis to suggest preventive guidelines for SCM in small-holder dairy cows.

## 2. Materials and Methods

### 2.1. Study Design, Data Collection, and Milk Sampling

#### 2.1.1. Study Design

This cross-sectional study conducted from April 2018 to November 2019 involved 40 private small-holder dairy farms from eight cities/counties in six districts of Gansu Province. The multi-stage and disproportionate stratified random sampling methods were used for sampling purposes in different dairy farming areas. The inclusion criteria for the dairy farm selection were a small-holder family farm, the availability of good quality records and data, interest in participating in this research for at least one year, an average herd size ranging from 10 to 100, and cows with no visible abnormality of their milk and udder. Cows in the first 14 days of lactation and those with any CM signs or symptoms were excluded from this study. The farms were divided into two strata (herds with 10–50 dairy cows and herds with 50–100 dairy cows), and then the simple random cows were selected within each stratum.

#### 2.1.2. Data Collection

During farm visits, farmers were interviewed by a member of the research team about the potential risk factors for mastitis at the herd and cow levels using a prepared questionnaire, which included questions on general farm data (e.g., farm management, herd size, feeding practices, and milk production) and individual cow details (parity, age, and lactation stage). The questionnaire also included questions on the milking order of cows with confirmed mastitis (subclinical or clinical), pre-and post-milking teat disinfection, and the use of gloves. In the present study, all 40 small-holder farms were visited one time during the survey period, and 530 Chinese Holstein dairy cows without any signs of clinical mastitis were in their 1st to 7th parity (categorized as 1–2 calves, 3–4 calves, 5–6 calves, and ≥7 calves). Three age categories were defined: 2–5, 6–8, and ≥9 years. Herd- and cow-level information, such as age, parity, lactation length (categorized as 1–3, 4–6, and 7–9 months), milk yield (categorized as ≥25 kg/day and <25 kg/day), teat lesions (regarded as yes or no), CM history (regarded as yes or no), technical training (regarded as yes or no), and udder cleanliness, was collected and recorded by a member of the research team based on farm owners’ responses and in some cases by abstracting the farm records.

#### 2.1.3. Milk Sampling

The number of milk samples collected from each city was as follows: Lanzhou (*n* = 80), Zhangye (*n* = 83), Linze (*n* = 68), Jiuquan (*n* = 80), Jinchang (*n* = 71), Tianzhu (*n* = 8), Tianshui (*n* = 76), and Baiyin (*n* = 64). All milking cows were checked for CM through udder observation, palpation, and changes in milk appearance. The screening of SCM was conducted using the California Mastitis Test (CMT) as used previously [18]. The milk samples were collected aseptically according to the guidelines of the National Mastitis Council [19]. The cows’ teats were cleaned with lukewarm water, dried with a clean towel, and disinfected. The first few streaks of milk were discarded, and then approximately 10 mL of milk per quarter was milked into a CMT paddle; a visual assessment of the milk was performed for consistency, color, and clots. CMT scores were recorded as zero (0) or trace (1) for negative and +2, +3, or +4 for positive. Each cow was evaluated for all udder quarters through the CMT. Cows with at least one quarter with a positive CMT score (CMT score ≥ 2) and no visible abnormality in the milk and udder were considered SCM-positive [18]. The cow and CMT levels were recorded for each SCM-positive cow, and 10 mL of milk was collected from a positive quarter in a sterile 15 mL tube and stored at 4 °C until it reached the laboratory. The milk samples were transported within 24 h to the microbiology laboratory at Lanzhou Institute of Husbandry and Pharmaceutical Sciences of the Chinese Academy of Agriculture Sciences, Lanzhou, China, for further examination. The samples were processed immediately for microbiological examination after reaching the laboratory.

### 2.2. Isolation and Identification of Bacterial Species

Each milk sample was inoculated separately onto 5% sheep blood agar (SBA, Beijing Solarbio Science and Technology Co. Ltd., Beijing, China), MacConkey agar (MCA, Huankai Science and Technology Co. Ltd., Guangzhou, Guangdong, China), and modified Edwards medium (EDM; Oxoid LTD, Basingstoke, Hampshire, UK) plates and incubated for 24–36 h at 37 °C. The bacterial species were identified based on growth characteristics, colony morphology, hemolysis pattern, Gram staining, and biochemical tests. Samples were considered culture-positive if one or more bacterial colonies were observed.

Among Gram-negative bacteria, *Escherichia coli* were differentiated from *Klebsiella* and other species by the colony morphology and biochemical tests (oxidase test, the H_2_S test, the triple sugar iron (TSI) test, the urease test, and the “IMViC” tests (Indole, Methyl-Red, Voges–Proskauer, and Citrate utilization)). The catalase test was used to distinguish *Staphylococcus* (catalase-positive) from *Streptococcus* (catalase-negative). *S. aureus* was distinguished from other staphylococci by *α*- and *β*-hemolysis on SBA plate, Gram staining, and other tests, including the Christie–Atkins–Munch-Petersen test (CAMP), tube coagulase test (using fresh rabbit plasma), and maltose fermentation test. For the suspected *Streptococcus* spp., the CAMP, aesculin hydrolysis, and sodium hippurate hydrolysis tests were performed, and *S. agalactiae* (CAMP-positive, aesculin-negative, and sodium hippurate-negative); *S. dysgalactiae* (CAMP-negative, aesculin-negative, and sodium hippurate-negative); and *S. uberis* (CAMP-negative, could hydrolyze aesculin and sodium hippurate) were found. Milk samples yielding two bacterial species were grouped as a mixed culture, whereas samples yielding three or more species were considered contaminated, following the guidelines of a previously published study [20].

### 2.3. Antimicrobial Susceptibility Testing

Antimicrobial susceptibility testing was conducted using the Kirby–Bauer disk diffusion method following the guidelines of the Clinical and Laboratory Standards Institute (CLSI) [21]. Antimicrobial agents were selected according to their availability in commercial intramammary infusion products. The antibiotics tested include penicillin (10 U), ampicillin (10 µg), amoxicillin–sulbactam (10 µg), ceftazidime (30 µg), streptomycin (10 µg), neomycin (30 µg), kanamycin (30 µg), gentamicin (10 µg), spectinomycin (100 µg), trimethoprim–sulfamethoxazole (SXT, 25 µg), norfloxacin (10 µg), ciprofloxacin (5 µg), vancomycin (30 µg), tetracycline (30 µg), doxycycline (30 µg), and erythromycin (15 µg) disks (Oxoid LTD, Basingstoke, Hampshire, UK). Pure cultures of six predominant isolated bacteria were selected (*n* = 211) and standardized to 0.5 MacFarland using DensiCHEK-plus (BioMerleux, Inc., Durham, NC, USA). Mueller–Hinton (MH) agar (MH agar, Oxoid LTD, Basingstoke, Hampshire, UK) plates were swabbed with standardized inoculums of the test organism (for Streptococcus species, 5% sheep defibrinated blood was added in MH agar). Antibiotic-impregnated paper disks were applied with a sterile dispenser, keeping at least a 24 mm distance between the disks. The plates were incubated at 37 °C for 20–24 h, and the diameter of the zone of inhibitions (ZOIs) around each disk was measured with the help of a calibrated Vernier Caliper and compared with standard breakpoints defined in CLSI [21] or European Committee on Antimicrobial Susceptibility Testing (EUCAST) [22] guidelines. Reference strains, *S. aureus* ATCC 29213 and *E. coli* ATCC 25922, were used as quality strains. Isolates that exhibited resistance to at least one antimicrobial agent from three different antimicrobial categories were recognized as multi-drug-resistant (MDR); isolates resistant to at least one agent in all antimicrobial categories and resistant to all agents in all antimicrobial categories were categorized as extensively drug-resistant (XDR) and pan-drug-resistant (PDR) isolates [1].

### 2.4. Statistical Analysis

At the cow level, the prevalence was expressed as the number of positive SCM cases over the total collected samples. The cow with a single quarter that was SCM-positive was considered a “case” and processed for risk factor analysis. The categorical data of risk factors were estimated using descriptive statistics. A continuity-corrected Chi-square test (Yates’ corrected χ^2^-test, for dichotomous variables, e.g., teat lesion, CM history, milk yield, and breeding training categories) or Fisher’s exact test (for polychotomous variables, e.g., age, parity, and lactation stage categories) for discrete data was used to identify and select the risk factors. Then, a logistic regression model was applied to estimate the potential risk factors associated with SCM episodes at a *p*-value of 0.20 using the forward-Wald’s test in SPSS software (version 25.0). All independent variables with significant associations (*p* < 0.20) for at least one level in the logistic regression analysis were selected as potential risk factors for multivariable analysis (multinomial logistic regression with backward stepwise elimination) based on maximum likelihood estimation (MLE). The Hosmer–Lemeshow χ^2^ statistic (for polychotomous variables) and deviance χ^2^ statistic (for dichotomous variables) in SPSS were used to assess the goodness-of-fit. The final significance level was *p* ≤ 0.01, and the confidence interval (C.I.) was 95%.

## 3. Results

### 3.1. Prevalence of SCM and Isolated Bacterial Species

**Figure 1:**
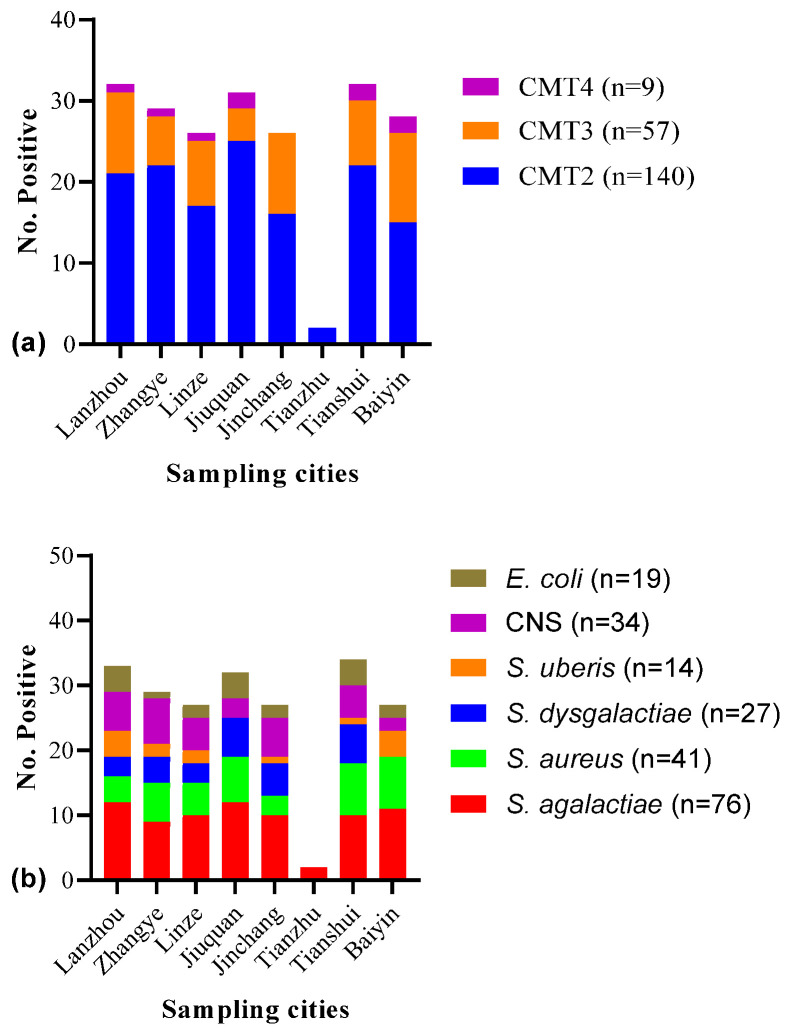
(**a**) Number of positive cases of subclinical mastitis (SCM) (*n* = 206) in small-holder dairy cows based on California Mastitis Test (CMT) scores. (**b**) Distribution of major SCM-associated bacteria (*n* = 211) isolated from milk of small-holder dairy cows.

Out of the 530 lactating cows and 2120 quarter milk samples examined, 38.87% (206/530, cow level) and 9.72% (206/2120, quarter level) were identified as positive for SCM by CMT screening, and all SCM-positive cows were identified as having infection in a single quarter/teat. The majority of the SCM-positive cows had CMT scores of 2 (140/206, 67.96%), followed by 3 (57/206, 27.67%) and 4 (9/206, 4.37%). The prevalence of SCM was noted to be higher in Lanzhou (32/206, 15.53%) and Tianshui (32/206, 15.53%), followed by Jiuquan (31/206, 15.05%), Zhangye (29/206, 14.08%), Baiyin (28/206, 13.59%), Linze and Jincahng (26/206, 12.62% each), and Tianzhu (2/206, 0.97%) (Figure 1a). Based on individual sample collection from each city, the prevalence of SCM was noted to be higher (43.75%) in Baiyin, followed by Tianshui (42.11%), Lanzhou (40.0%), Jiuquan (38.75%), Linze (38.24%), Jinchang (36.62%), Zhangye (34.94%), and Tianzhu (25.0%) with no significant difference (*p* > 0.05) between or within cities (Table 1).

SCM-positive samples, *n* = 206, were cultured on different microbiological media and checked for colony characteristics. Out of the total, *n* = 14 (14/206, 6.80%) milk samples had more than three different bacterial colony types been defined as contaminated and excluded from this study for further analysis. The remaining 193 milk samples yielded single (175/193, 90.67%) and double (18/193; 9.32%) colony types with 211 bacterial strains. Among the isolated bacterial species, *Streptococcus* and *Staphylococcus* were identified as predominant, accounting for 55.45% (117/211) and 35.55% (75/211), respectively. The distribution of individual species was *S. agalactiae* (36.02%, 76/211), followed by *S. aureus* (19.43%, 41/211), CNS (16.11%, 34/211), *S. dysgalactiae* (12.80%, 27/211), *E. coli* (9.00%, 19/21), and *S. uberis* (6.64%, 14/211). It was noted that contagious pathogens (55.45%, 117/211) predominated over environmental pathogens (44.55%, 94/211). The predominant pathogens in each sampling city were as follows: *S. agalactiae* in Lanzhou and Jiuquan; *S. aureus* in Baiyin and Tianshui; *S. dysgalactiae* in Jiuquan and Tianshui; *S. uberis* in Lanzhou and Baiyin; CNS in Zhangye; and *E. coli* in Lanzhou, Jiuquan, and Tianshui (Figure 1b).

### 3.2. Antimicrobial Susceptibility of Isolated Bacterial Species

**Figure 2:**
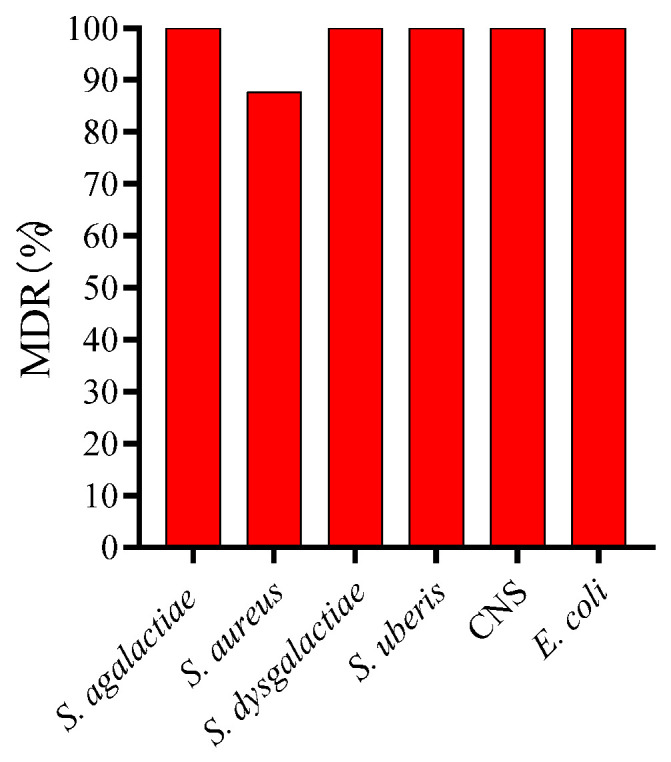
Percentage of multi-drug resistant (MDR) bacterial strains isolated from milk of small-holder dairy cows with SCM in Gansu Province, China.

The isolated bacterial species showed higher susceptibility to ampicillin, amoxicillin–sulbactam, ceftazidime, neomycin, kanamycin (except for *E. coli*), spectinomycin, norfloxacin, ciprofloxacin, and doxycycline at a rate of 73.17% to 100%. Moreover, *S. aureus* and CNS were >80% susceptible to gentamicin and tetracycline (Table 2). However, the isolates presented higher resistance to penicillin, streptomycin, gentamicin (except *S. aureus* and CNS), trimethoprim-sulfamethoxazole (SXT), vancomycin, and erythromycin, ranging from 57.89% to 100%. It was noted that *S. agalactiae*, *S. dysgalactiae*, and *S. uberis* were 100% sensitive to neomycin, kanamycin, spectinomycin, ciprofloxacin, and tetracycline, while sensitivity to ampicillin, amoxicillin–sulbactam, ceftazidime, norfloxacin, and doxycycline ranged from 78.57% to 100%. Similarly, *S. aureus* also exhibited higher sensitivity to ampicillin, amoxicillin-sulbactam, ceftazidime, neomycin, kanamycin, gentamicin, spectinomycin, norfloxacin, ciprofloxacin, tetracycline, and doxycycline (80.48–95.12%). CNS was found to be susceptible (100%) to amoxicillin–sulbactam, ceftazidime, gentamicin, spectinomycin, norfloxacin, ciprofloxacin, tetracycline, and doxycycline, while sensitivity to ampicillin, neomycin, and kanamycin ranged from 88.24 to 91.18%. The *E. coli* strains showed higher sensitivity to norfloxacin (100%) and ciprofloxacin (100%), followed by ceftazidime, doxycycline, neomycin, spectinomycin, tetracycline, ampicillin, and amoxicillin-sulbactam (73.68–94.74%). All isolated bacterial strains were 100% MDR except *S. aureus*, which was found to be 87.8% MDR (Figure 2). However, this study did not identify XDR or PDR strains.

### 3.3. Risk Factor Analysis

The cow with a single quarter that was SCM-positive was considered a “case” and processed for risk factor analysis. Among the eight variables analyzed by bivariate analysis, ≥9 years of age (OR = 5.146, C.I. = 2.511–10.546), ≥7th parity cow (OR = 7.022, C.I. = 2.739–18.002), previous CM history (OR = 3.946, C.I. = 1.837–8.476), and lesion on teats (OR = 3.199, C.I. = 1.721–5.946) exhibited strong association (*p* < 0.001) with SCM cases. It was noted that animals with an age ≥9 years had a 5.146 times increased risk as compared to animals in the 2–5- and 6–8-year age groups; animals with a ≥9 parity number had a 7.022 times increased risk as compared to animals in the 1–2 and 3–4 parity number groups. Similarly, a previous history of CM put animals at a 3.946 greater risk than those with no previous history of CM. Having a lesion on teats exposed animals to a 3.199 greater risk to SCM than those with no teat lesions (Table 3). Other potential factors found to be associated with increased SCM cases (*p* < 0.05) were cows in the 5–6th parity (OR = 2.627, C.I. = 1.127–6.124), at 7–9 months of lactation (OR = 1.814, C.I. = 1.147–2.870), and having an increased milk yield (OR = 1.677, 1.178–2.387). However, the *p*-value of all variables was noted to be ≤0.2 and considered for multivariate analysis to evaluate the independent effect of each variable. The multivariate analysis showed that age (6–8 years: OR = 2.746, C.I. = 1.756–4.293; ≥9 years: OR = 5.146, C.I. = 2.511–10.546), ≥3rd parity (OR = 2.673–7.022), a lactation period of 7–9 months (OR = 1.814, C.I. = 1.147–2.870), a previous history of CM (OR = 3.946, C.I. = 1.837–8.476), a teat lesion (OR = 3.199, C.I. = 1.721–5.946), ≥25 kg/d milk yield (OR = 0.596, C.I. = 0.419–0.849), and milking training (OR = 0.653, C.I. = 0.405–1.053) were potential risk factors (*p* ≤ 0.01) associated with SCM in small-holder dairy cows. Udder cleanliness and a lactation period of 4–6 months were not found to be potential risk factors (*p* ≥ 0.01) (Table 4).

In these analyses, the Hosmer–Lemeshow χ^2^ statistic was 0.000 with 1 or 2 df, *p* = 1.000 (for variables of age, parity, and lactation months), while the deviance χ^2^ statistic for teat lesions was 9.917 with 14 df, *p* = 0.768; that for CM history was 19.066 with 14 df, *p* = 0.162; that for milk yield was 20.019 with 14 df, *p* = 0.130; that for breeding training was 36.843 with 14 df, *p* = 0.001; and that for udder cleanliness was 32.992 with 14 df, *p* = 0.003, indicating a good model fit and suggesting appropriate model selection (based on goodness of model fit: χ^2^ statistic, *p* > 0.05), indicating that the difference between the predicted and observed values of the model is not statistically significant. The results of the binary logistic regression and final multinomial logistic regression are summarized in Table 3 and Table 4, respectively.

## 4. Discussion

Mastitis is a significant disease in dairy cows which causes substantial economic losses in terms of higher treatment cost and decreased milk production. The dairy industry has become well established in China in recent years at the commercial scale as well as a small scale. Previously, multiple studies were conducted to assess the prevalence of clinical and subclinical mastitis in the commercial dairy industry [11,23,24]. So far, the assessment of SCM in small-holder dairy cows, especially in Gansu Province, China, is lacking, which may compromise the knowledge of potential strategies to prevent and control this disease. The present study focused on the prevalence of SCM in small-holder dairy cows in Gansu Province, China, along with the identification and antimicrobial susceptibility of predominant bacterial pathogens associated with SCM. Moreover, this study also analyzed the potential risk factors associated with SCM in small-holder dairy cows through bivariate and multivariate regression analyses.

The present study noted an overall 38.87% prevalence of SCM in small-holder dairy herds, which is approximately (37.7%) in accordance with that reported by Chen et al. [11] in China from 2012 to 2021. The global SCM prevalence was reported to be 42% by Krishnamoorthy et al. [25] in their systematic review and meta-analysis, which is also noted to be close to the prevalence obtained in our study. However, SCM prevalence in South Asian countries, including Pakistan, Sri Lanka, India, and Bangladesh, varies from 20 to 80% with an average of 50% [26], which is a little higher than that obtained in the current study. The difference in the prevalence of SCM may be due to farming type, geographical location, animal density, hygiene and sanitation practices, and other potential risk factors [1]. High SCM prevalence in small-holder dairy herds can be attributed to poor hygiene, poor bedding and housing, bad milking practices, and other cow-level factors such as age, parity, and lactation.

The present study identified *S. agalactiae*, *S. aureus*, CNS, *S. dysgalactiae*, *E. coli*, and *S. uberis* as predominant bacterial pathogens associated with SCM. These findings are in accordance with multiple previous studies conducted in China and globally that identified *S. aureus* [1,23,24,27], *S. agalactiae* [24,27,28,29], CNS [24,27], *S. dysgalactiae* [27,30,31], *E. coli* [11,24,27,32], and *S. uberis* [27,30] as predominant bacterial species associated with SCM. However, there are differences in the prevalence of these pathogens in the current study and previous studies. The methodology used in the current and previous studies may vary, which can also be a possible reason for the differences and can make it challenging to compare prevalence among regions and countries. *S. aureus* and *S. agalactiae* are known to be highly contagious pathogens responsible for SCM. They can reside for a longer period in the mammary glands through the evasion of the host immune system and the invasion of deeper tissue because of the production of multiple enzymes and toxins [6,15]. Both can be transmitted to healthy cows through poor milking hygiene, contaminated utensils, milking machines, and milkers’ hands [15]. *S. uberis*, *S. dysgalactiae*, and *E. coli* are considered environmental pathogens that can be transmitted through poor farm cleanliness and sanitation, inadequate manure removal, and bedding material [33]. The presence of these pathogens in cow milk with the hidden form of mastitis may be a possible threat, i.e., increased AMR and infection, to human health through the consumption of contaminated milk [34]. Therefore, the present study recommends that small-holder and large-scale dairy farms pay immediate attention to this matter and implement control measures to prevent the spread of SCM in dairy cows and their significant impacts on public health.

The antibiotic susceptibility of the recovered bacterial species demonstrated the presence of 100% MDR strains, except *S. aureus*, which accounted for 87.8%, and no XDR or PDR isolates were identified. A similar study conducted in Bangladesh reported that isolates were 98.16% MDR *S. aureus*, and no XDR and PDR isolates were recovered [1], except *K. pneumoniae* (23.4% XDR), which was not isolated in the current study. Over the study period, the six pathogens showed higher susceptibility to ampicillin, amoxicillin–sulbactam, ceftazidime, neomycin, kanamycin, spectinomycin, norfloxacin, ciprofloxacin, tetracycline, and doxycycline (ranging from 73.17% to 100.00%). Similar findings were presented by Sweeney et al. [35], who noted the increased sensitivity of ampicillin and ceftiofur against *S. dysgalactiae*, *S. uberis*, *S. aureus*, and *E. coli* isolated from North American cattle. Similar findings were presented by Emon et al. [1], who observed the increased susceptibility (100%) of *E. coli* to ciprofloxacin. Similarly, Abed et al. [15] also presented the increased susceptibility of *S. aureus* and NAS to ciprofloxacin and the increased resistance (88%) of *E. coli* to SXT. In contrast, Abed et al. [15] noted the increased resistance of *E. coli* to doxycycline (90%) and ciprofloxacin (84%). The increased susceptibility to these antibiotics may suggest that these antibiotics be used in the prevention and treatment of SCM in the studied area. In addition, the six pathogens showed higher resistance rates against penicillin, streptomycin, gentamicin, trimethoprim-sulfamethoxazole (SXT), vancomycin, and erythromycin. A study conducted in Bangladesh also identified the increased resistance of *E. coli* to gentamicin and streptomycin [36]. Another study conducted by Ren et al. [23] in Xinjiang, China, isolated *S. aureus* from SCM cows who also presented increased resistance to penicillin, gentamicin, and erythromycin. The increased AMR might be due to the long-term and unjustified use of these antibiotics in mastitis treatment, which potentially led to antibiotic selection pressure exerted on bacteria and multi-drug-resistant strains [37]. The genetic basis of the emergence of AMR is the horizontal gene transfer (HGT) of drug-resistance genes in bacteria, which can lead to the emergence of multi-drug-resistant strains such as extended-spectrum β–β-lactamase-producing strains [38] and methicillin-resistant *S. aureus* [39]. Therefore, there is a need to study the genetic basis of AMR by, for example, identifying resistance genes through PCR or whole-genome sequencing and their dissemination through HGT mechanisms in isolated bacterial species. The probability of the successful treatment of mastitis depends on the immune status of the cow, the virulence characteristics of the pathogen, and the efficacy of the treatment protocol [40].

Invasive bacterial infections are the predominant cause of bovine mastitis. Therefore, prompt treatment with effective antimicrobials is especially important to reduce the risk of SCM and is commonly implemented for mastitis prevention and control. However, the failure of antimicrobial treatment is common; the World Health Organization has stated that any use of antimicrobial agents is associated with the risk of inducing resistance to antimicrobial agents among bacteria [41]. Kamel and Bakry [42] demonstrated that more than 70% of the total antibiotics used were for subclinical/clinical mastitis control and treatment. Moreover, Kovačević et al. [43] reported that antimicrobial use was positively associated with AMR in bovine mastitis pathogens. In the present study, we identified antimicrobial sensitivity/resistance profiles for the six most frequently isolated predominant pathogens (five Gram-positive cocci and one Gram-negative bacillus), commonly used in veterinary and human medicine, against 16 antimicrobial agents in small-holder dairy farms in Gansu Province between 2018 and 2019. Antimicrobial therapy is a valuable tool used to control subclinical and clinical mastitis in dairy cows, and it is important to monitor the profiles of antimicrobial susceptibility and resistance patterns, which would help to optimize the correct choice of antimicrobial for treatment purposes.

The current study showed an increased risk of SCM with increased cow age (≥6 years), parity (≥3rd), and lactation month (7–9), which was consistent with the results of many other studies conducted earlier in China and globally [11,26,27]. Increased age and parity may be associated with an increased frequency and duration of milking which can decrease immunity and increase the chances of udder damage and bacterial infections [26]. Moreover, this is a time of high milk production for cows, and pathogens (e.g., *S. agalactiae*) can easily invade the udder, resulting in an increased incidence of mastitis [44]; thus, cows aged ≥6 years old had at least one quarter that suffered from mastitis. However, some studies have reported a downward trend in the prevalence of SCM in cows over 8 years of age, which is inconsistent with the results of this study, possibly because the milk yield of cows in this age range decreases gradually, and the burden on the udder is also reduced [45].

Furthermore, our findings demonstrated that SCM was positively associated with teat lesions as well as clinical mastitis history (*p* < 0.01), in agreement with the results reported previously in China and other countries [44,46]. Moreover, the misuse of milking apparatuses can be associated with increased teat lesions, which increases the incidence of mastitis by promoting udder infections [47]. In addition, we observed that some dairy cows in small-holder farms were housed under poorer sanitary conditions than cows on large-scale intensive dairy farms, which might contribute to the high incidence of mastitis; these results are consistent with the findings reported from developing countries [2,7,46]. The present study also recognized that increased milk yield and no milking training were strongly associated with SCM cases in small-holder dairy cows, which is consistent with previous studies [48,49] that report that increased milk yield can result in a higher risk of mastitis. Increased milk yield is directly correlated with teat canal diameter and stretching ability. The teat canal remains open for a longer period in cows with a higher milk yield compared to low-milk-yield cows, which can result in increased chances of the penetration of environmental pathogens such as *E. coli* and can put cows at an increased risk of SCM [48]. The significant association of milk training with SCM indicates the importance of milking methods such as full-hand milking, and the proper handling of milking machines can result in lower chances of SCM [26,50] compared to those when using different methods. The present study noted that small-holding dairy farmers milked their cows using improper hand-milking techniques and seldom used machines, which may be a possible reason for the increased risk of SCM.

The prevalence rate of SCM among small-holder dairy cows was noted to be higher, which indicates that mastitis is still a significant problem in small-holder dairy herds. During this investigation, most of the small-holder farmers were unaware of the SCM cases; in particular, there was a lack of knowledge regarding mastitis and infection control precautions that could be taken in their herds. The present study recommends increasing the awareness and knowledge of mastitis through the implementation of activities, including a better understanding of field mastitis diagnoses, the causes of antimicrobial resistance and its consequences, prevention and control strategies for mastitis, and potential risk factors associated with SCM and CM.

## 5. Conclusions

The current study elucidates SCM prevalence, predominant bacterial pathogens, their antimicrobial susceptibility profiles, and potential risk factors associated with SCM in small-holder dairy cows in Gansu Province, China. The findings of the present study revealed a 38.87% cow-level and 9.72% quarter-level prevalence of SCM. The predominant bacterial pathogens isolated were *S. agalactiae* (36.02%), *S. aureus* (19.43%), CNS (16.11%), *S. dysgalactiae* (12.80%), *E. coli* (8.59%), and *S. uberis* (6.64%). Antimicrobial susceptibility profiles revealed the increased resistance (>85%) of these pathogens to penicillin, streptomycin, trimethoprim-sulfamethoxazole, vancomycin, and erythromycin. Moreover, the possible chances of developing clinical mastitis were also highlighted. Moreover, *S. agalactiae*, *S. dysgalactiae*, and *S. uberis* also exhibited increased resistance (>77%) to gentamicin and tetracycline. The risk factor analysis revealed that older age, higher parity, late lactation month, higher milk yield, previous history of CM, lesion on teats, and no milking training were the potential risk factors associated with SCM in small-holder dairy herds in the studied population during the study period. These findings highlight the need for antimicrobial stewardship programs and effective control strategies to stop the spread of SCM in healthy cows and their potential impacts on human and animal health. These strategies may include the routine surveillance of antimicrobial resistance, the training of small-scale farmers, and the education of them about potential risk factors, preventive strategies, and antimicrobial stewardship. This study also recommends that molecular investigations be conducted, such as the identification of antibiotic resistance genes and their dissemination mechanisms, to stop their spread within and between bacterial species.

## Figures and Tables

**Table 1:** Area-wise prevalence of SCM in small-holder dairy cows in eight counties of Gansu Province, China.

Sampling Areas	No. of Samples	No. of SCM-Positive Samples	% Age of SCM-Positive Samples	C.I. (95%)	*p*-Value
Lanzhou	80	32	40.0 ^a^	29.39–51.58	0.936
Zhangye	83	29	34.94 ^a^	25.02–46.27	
Linze	68	26	38.24 ^a^	26.96–50.86	
Jiuquan	80	31	38.75 ^a^	28.26–50.33	
Jinchang	71	26	36.62 ^a^	25.75–48.95	
Tianzhu	08	02	25.0 ^a^	4.450–64.42	
Tianshui	76	32	42.11 ^a^	31.05–53.97	
Baiyin	64	28	43.75 ^a^	31.58–56.67	
Total	530	206	38.87 ^a^	34.72–43.18	

C.I. = confidence interval; *p*-value ≥ 0.05 indicates a non-significant difference. Similar superscripts in columns indicate non-significant differences within different sampling areas.

**Table 2:** Antimicrobial susceptibility of bacterial species isolated from milk of small-holder dairy cows with SCM in Gansu Province, China.

Antimicrobial Agent/Disk Content (µg or U)	Bacterial Species																	
	*S. agalactiae* (*n* = 76)			*S. aureus* (*n* = 41)			*S. dysgalactiae* (*n* = 27)			*S. uberis* (*n* = 14)			CNS (*n* = 34)			*E. coli* (*n* = 19)		
	R, No. (%)	I, No. (%)	S, No. (%)	R, No. (%)	I, No. (%)	S, No. (%)	R, No. (%)	I, No. (%)	S, No. (%)	R, No. (%)	I, No. (%)	S, No. (%)	R, No. (%)	I, No. (%)	S, No. (%)	R, No. (%)	I, No. (%)	S, No. (%)
Penicillin (10 U)	76 (100.00)	0 (0.0)	0 (0.0)	36 (87.80)	0 (0.0)	5 (12.20)	27 (100.00)	0 (0.0)	0 (0.0)	14 (100.00)	0 (0.0)	0 (0.0)	32 (94.12)	2 (5.88)	0 (0.0)	19 (100.00)	0 (0.0)	0 (0.0)
Ampicillin (10 µg)	0 (0.0)	0 (0.0)	76 (100.00)	2 (4.88)	9 (21.95)	30 (73.17)	4 (14.81)	0 (0.0)	23 (85.19)	0 (0.0)	0 (0.0)	14 (100.00)	0 (0.0)	4 (11.76)	30 (88.24)	0 (0.0)	4 (21.05)	15 (78.95)
Amoxicillin– Sulbactam (10 µg)	0 (0.0)	8 (10.53)	68 (89.47)	0 (0.0)	5 (12.2)	36 (87.80)	0 (0.0)	0 (0.0)	27 (100.00)	0 (0.0)	0 (0.0)	14 (100.00)	0 (0.0)	0 (0.0)	34 (100.00)	0 (0.0)	5 (26.32)	14 (73.68)
Ceftazidime (30 µg)	0 (0.0)	0 (0.0)	76 (100.00)	0 (0.0)	4 (9.76)	37 (90.24)	0 (0.0)	3 (11.11)	24 (88.89)	0 (0.0)	2 (14.29)	12 (85.71)	0 (0.0)	0 (0.0)	34 (100.00)	0 (0.0)	1 (5.26)	18 (94.74)
Streptomycin (10 µg)	76 (100.00)	0 (0.0)	0 (0.0)	41 (100.00)	0 (0.0)	0 (0.0)	27 (100.00)	0 (0.0)	0 (0.0)	14 (100.00)	0 (0.0)	0 (0.0)	34 (100.00)	0 (0.0)	0 (0.0)	17 (89.47)	0 (0.0)	2 (10.53)
Neomycin (30 µg)	0 (0.0)	0 (0.0)	76 (100.00)	0 (0.0)	2 (4.88)	39 (95.12)	0 (0.0)	0 (0.0)	27 (100.00)	0 (0.0)	0 (0.0)	14 (100.00)	0 (0.0)	3 (8.82)	31 (91.18)	0 (0.0)	2 (10.53)	17 (89.47)
Kanamycin (30 µg)	0 (0.0)	0 (0.0)	76 (100.00)	3 (7.32)	5 (12.2)	33 (80.49)	0 (0.0)	0 (0.0)	27 (100.00)	0 (0.0)	0 (0.0)	14 (100.00)	0 (0.0)	4 (11.76)	30 (88.24)	4 (21.05)	2 (10.53)	13 (68.42)
Gentamicin (10 µg)	70 (92.11)	6 (7.89)	0 (0.0)	7 (17.07)	0 (0.0)	34 (82.93)	21 (77.78)	3 (11.11)	3 (11.11)	12 (85.71)	0 (0.0)	2 (14.29)	0 (0.0)	0 (0.0)	34 (100.00)	11 (57.89)	0 (0.0)	8 (42.11)
Spectinomycin (100 µg)	0 (0.0)	0 (0.0)	76 (100.00)	0 (0.0)	3 (7.32)	38 (92.68)	0 (0.0)	0 (0.0)	27 (100.00)	0 (0.0)	0 (0.0)	14 (100.00)	0 (0.0)	0 (0.0)	34 (100.00)	0 (0.0)	2 (10.53)	17 (89.47)
SXT (25 µg)	76 (100.00)	0 (0.0)	0 (0.0)	41 (100.00)	0 (0.0)	0 (0.0)	27 (100.00)	0 (0.0)	0 (0.0)	14 (100.00)	0 (0.0)	0 (0.0)	30 (88.24)	0 (0.0)	4 (11.76)	19 (100.00)	0 (0.0)	0 (0.0)
Norfloxacin (10 µg)	0 (0.0)	7 (9.22)	69 (90.78)	0 (0.0)	3 (7.32)	38 (92.68)	0 (0.0)	3 (11.11)	24 (88.89)	0 (0.0)	3 (21.43)	11 (78.57)	0 (0.0)	0 (0.0)	34 (100.00)	0 (0.0)	0 (0.0)	19 (100.00)
Ciprofloxacin (5 µg)	0 (0.0)	0 (0.0)	76 (100.00)	0 (0.0)	2 (4.88)	39 (95.12)	0 (0.0)	0 (0.0)	27 (100.00)	0 (0.0)	0 (0.0)	14 (100.00)	0 (0.0)	0 (0.0)	34 (100.00)	0 (0.0)	0 (0.0)	19 (100.00)
Vancomycin (30 µg)	71 (93.42)	5 (6.58)	0 (0.0)	35 (85.37)	0 (0.0)	6 (14.63)	27 (100.00)	0 (0.0)	0 (0.0)	13 (92.86)	0 (0.0)	1 (7.14)	31 (91.18)	3 (8.82)	0 (0.0)	19 (100.00)	0 (0.0)	0 (0.0)
Tetracycline (30 µg)	76 (100.00)	0 (0.0)	0 (0.0)	2 (4.88)	6 (19.52)	33 (80.48)	27 (100.00)	0 (0.0)	0 (0.0)	14 (100.00)	0 (0.0)	0 (0.0)	0 (0.0)	0 (0.0)	34 (100.00)	0 (0.0)	3 (15.79)	16 (84.21)
Doxycycline (30 µg)	0 (0.0)	8 (10.53)	68 (89.47)	0 (0.0)	4 (9.76)	37 (90.24)	0 (0.0)	0 (0.0)	27 (100.00)	0 (0.0)	0 (0.0)	14 (100.00)	0 (0.0)	0 (0.0)	34 (100.00)	0 (0.0)	1 (5.26)	18 (94.74)
Erythromycin (15 µg)	76 (100.00)	0 (0.0)	0 (0.0)	35 (85.37)	2 (4.88)	4 (9.76)	27 (100.00)	0 (0.0)	0 (0.0)	14 (100.00)	0 (0.0)	0 (0.0)	29 (85.29)	0 (0.0)	5 (14.71)	17 (89.47)	0 (0.0)	2 (10.53)

CNS, coagulase-negative staphylococci; SXT, trimethoprim–sulfamethoxazole; NA = no breakpoint; S, susceptible; I, intermediate; R, resistant. Susceptible breakpoints (mm, zone diameter) were defined based on CLSI (2013, 2017) or EUCAST (2015).

**Table 3:** Bivariable analysis of potential risk factors associated with SCM in small-holder dairy cows based on CMT screening.

Variables	Total	CMT		Binary Logistic Regression Analysis						
		Positive (*n* = 206), No. (%)	Negative (*n* = 324) No. (%)	B	S.E.	Wald	df	*p*-Value	O.R.	95% C.I.
Age (years)										
2–5	146	32 (15.5)	114 (35.2)						1	
6–8	340	148 (71.9)	192 (59.3)	0.628	0.326	3.721	1	0.054	1.871	0.990–3.547
≥9	44	26 (12.6)	18(5.5)	1.638	0.366	20.02	1	0.000	5.146	2.511–10.546
Parity										
1–2	97	18 (8.7)	79 (24.4)						1	
3–4	177	67 (32.5)	110 (34.0)	0.644	0.424	2.306	1	0.129	1.905	0.829–4.375
5–6	230	105 (51.0)	125 (38.6)	0.966	0.432	5.001	1	0.025	2.627	1.127–6.124
≥7	26	16 (7.8)	10 (3.0)	1.949	0.480	16.46	1	0.000	7.022	2.739–18.002
Lactation months										
1–3	168	55 (26.7)	113 (34.9)						1	
4–6	217	83 (40.3)	134 (41.3)	0.355	0.217	2.665	1	0.103	1.426	0.931–2.183
7–9	145	68 (33.0)	77 (23.8)	0.596	0.234	6.486	1	0.011	1.814	1.147–2.870
Teat lesion										
Absent	482	175 (85.0)	307 (94.8)						1	
Present	48	31 (15.0)	17 (5.2)	1.163	0.316	13.51	1	0.000	3.199	1.721–5.946
CM history										
No	497	183 (88.8)	314 (96.9)						1	
Yes	33	23 (11.2)	10 (3.1)	1.373	0.390	12.38	1	0.000	3.946	1.837–8.476
Milk yield (kg/d)										
<25.0	229	105 (51.0)	124 (38.3)						1	
≥25.0	301	101 (49.0)	200 (61.7)	0.517	0.180	8.223	1	0.004	1.677	1.178–2.387
Milking training										
Yes	436	177 (85.9)	259 (79.9)						1	
No	94	29 (14.1)	65 (20.1)	0.426	0.244	3.062	1	0.080	1.532	0.950–2.469
Udder cleanliness										
Yes	395	144 (69.9)	251 (77.5)						1	
No	115	62 (30.1)	73 (22.5)	0.392	0.202	3.776	1	0.052	1.480	0.997–2.199

No. = number; O.R. = Odds Ratio; C.I. = confidence interval; Absent = teat not damaged; Present = teat injured.

**Table 4:** Final logistic regression model of potential risk factors associated with SCM in small-holder dairy cows.

Risk Factors	Multivariate Analysis						
	B	S.E.	Wald	df	*p*-Value	O.R.	95% C.I.
Age (years)							
2–5						1	
6–8	1.010	0.228	19.629	1	0.000	2.746	1.756–4.293
≥9	1.638	0.366	20.021	1	0.000	5.146	2.511–10.546
Parity							
1–2						1	
3–4	0.983	0.304	10.483	1	0.001	2.673	1.474–4.848
5–6	1.305	0.293	19.855	1	0.000	3.687	2.077–6.544
≥7	1.949	0.480	16.466	1	0.000	7.022	2.739–18.002
Lactation months							
1–3						1	
4–6	0.241	0.216	1.248	1	0.264	1.273	0.834–1.942
7–9	0.596	0.234	6.486	1	0.011	1.814	1.147–2.870
Teat lesion							
Absent						1	
Present	1.163	0.316	13.515	1	0.000	3.199	1.721–5.946
CM history							
No						1	
Yes	1.373	0.390	12.388	1	0.000	3.946	1.837–8.476
Milk yield (kg/d)							
<25.0						Reference	
≥25.0	−0.517	0.180	8.223	1	0.004	0.596	0.419–0.849
Milking training							
Yes						1	
No	−0.426	0.244	3.062	1	0.000	0.653	0.405–1.053
Udder cleanliness							
Yes						1	
No	0.392	0.202	3.776	1	0.052	1.480	0.997–2.199

O.R. = Odds Ratio; C.I. = confidence interval; Absent = teat not damaged; Present = teat injured.

## Data Availability

The original contributions presented in this study are included in the article/Supplementary Material. Further inquiries can be directed to the corresponding author.

## Supplementary Materials

The following supporting information can be downloaded at https://www.mdpi.com/article/10.3390/microorganisms12122643/s1, Table S1: Questionnaire data collected from Lanzhou; Table S2: Questionnaire data collected from Zhangye; Table S3: Questionnaire data collected from Linze; Table S4: Questionnaire data collected from Jiuquan; Table S5: Questionnaire data collected from Jinchang; Table S6: Questionnaire data collected from Tianzhu; Table S7: Questionnaire data collected from Tianshui; Table S8: Questionnaire data collected from Baiyin.
